# Parental Stress, Maternal Health, and Children’s Vision-Related Quality of Life in Total Childhood Blindness: A Cross-Sectional Study

**DOI:** 10.3390/ijerph23020162

**Published:** 2026-01-28

**Authors:** Julio Cesar Souza-Silva, Viviane Matias da Costa Souza, Thallita de Freitas Ramos, Cleusa Alves Martins, Edinamar Aparecida Santos da Silva, Marco Túlio Antônio Garciazapata, Milton Ruiz Alves, Maria Alves Barbosa

**Affiliations:** 1 Department of Ophthalmology and Otorhinolaryngology, Faculty of Medicine, University of São Paulo, São Paulo Campus, University of São Paulo (USP), São Paulo 05409-000, Brazil; 2 School of Psychology, Estácio de Sá University, Goiânia 74063-010, Brazil; draviviane.psi@hotmail.com; 3 University Unit of Ceres, State University of Goiás, Ceres 76300-000, Brazil; thallita.ramos@ueg.br; 4 School of Nursing, Federal University of Goiás (UFG), Goiânia 74605-080, Brazil; cleusa.alves@gmail.com (C.A.M.); edinamar@ufg.br (E.A.S.d.S.); maria_barbosa_alves@ufg.br (M.A.B.); 5 Institute of Tropical Pathology and Public Health, Department of Tropical Medicine and Dermatology, Federal University of Goiás (UFG), Goiânia 74605-050, Brazil; zapata@ufg.br; 6 Hospital das Clínicas, Faculty of Medicine, University of São Paulo (HC-FMUSP), São Paulo 05409-000, Brazil; milton.alves@hc.fm.usp.br

**Keywords:** parental stress, childhood blindness, congenital blindness, functional vision, pediatric ophthalmology, maternal health, caregivers, early intervention

## Abstract

**Highlights:**

**Public health relevance—How does this work relate to a public health issue?**

- Total childhood blindness is a rare but severe condition with lifelong developmental, functional, and psychosocial consequences for affected children and their families.
- Families of children with total blindness often live in conditions of socioeconomic vulnerability and limited access to specialized rehabilitation and psychosocial support.

**Public health significance—Why is this work of significance to public health?**

- This study shows high levels of parenting stress and maternal health symptoms among mothers of totally blind children in Brazil, alongside markedly impaired vision-related quality of life in their children.
- The findings highlight that parental stress, maternal health, and children’s functional vision are closely intertwined, especially in socially vulnerable contexts, and should be addressed together in public health strategies.

**Public health implications—What are the key implications or messages for practitioners, policy makers and/or researchers in public health?**

- Integrating systematic screening for parenting stress and maternal mental health into pediatric ophthalmology and rehabilitation services could help identify families at higher risk and guide targeted support.
- Public health policies should prioritize multidisciplinary, family-centered care—including psychological, social, and rehabilitative interventions—to reduce parental burden and improve the quality of life of children with total blindness.

**Abstract:**

Parental stress is a critical yet understudied dimension of childhood total blindness, a condition that imposes substantial developmental, emotional, and functional challenges on families. This cross-sectional study assessed parenting stress, maternal health symptoms, and children’s functional vision-related quality of life in 81 mothers of children aged 0 to 12 years with total congenital blindness. Parenting stress was assessed in the full sample using the Parenting Stress Index–Fourth Edition (PSI-4). Children’s functional vision-related quality of life was evaluated in age-specific subsamples using the Quality of Family Vision Impact (QFVI-3 for children aged 0–3 years and QFVI-7 for children aged 3–7 years). All participants also completed a sociodemographic and maternal health survey. Total Parent Stress showed moderately elevated percentile scores (mean ≈ 67), with the highest PSI-4 subdomains in Adaptability, Depression, and Health. Approximately 21% of mothers scored within the clinical range for high stress. Maternal symptoms including sadness, insomnia, headaches, forgetfulness, and musculoskeletal pain were significant (all *p* < 0.01). QFVI global scores indicated moderate impairments in functional vision-related quality of life across age groups. Life Stress demonstrated a small-to-moderate negative correlation with QFVI-7, suggesting that cumulative environmental stressors may adversely affect children’s functional outcomes. Several factors were associated with more favorable outcomes. Among children under three years of age, maternal engagement in physical activity was associated with higher QFVI scores, whereas among children aged 3–7 years, school attendance was associated with higher functional vision-related quality of life scores. In contrast, sociodemographic disadvantage, limited access to educational adaptations, and reduced maternal participation in work or leisure activities were associated with higher levels of parental stress. These findings highlight the importance of multidisciplinary, family-centered care incorporating psychosocial assessment, early stimulation, orientation and mobility support, and maternal mental health interventions in pediatric ophthalmology.

## 1. Introduction

Childhood blindness is a rare but severe condition with lifelong developmental, social, emotional, and functional consequences for affected children and their families. Global estimates indicate that approximately 500,000 children become blind each year, with a prevalence ranging from 0.3 to 1.5 per 1000 depending on socioeconomic context [1]. In Brazil, childhood blindness affects an estimated 0.5–0.6 per 1000 children, corresponding to approximately 22,000 to 26,000 individuals living with severe visual impairment [2]. Total blindness—defined as the absence of light perception and classified as WHO Category 5—results in profound limitations in motor development, communication, mobility, learning, and psychosocial functioning [1]. Because childhood blindness affects many decades of life, and nearly half of cases worldwide are considered preventable, it is recognized as a global public health priority [1,2].

Mothers are typically the primary caregivers for children with total blindness and face complex demands involving daily care, environmental adaptation, coordination of medical and rehabilitation services, and support for social and educational inclusion. These responsibilities often occur within contexts marked by socioeconomic vulnerability and limited access to specialized services. Stress, as first described by Selye as a nonspecific physiological response to internal or external demands [3], becomes chronic when caregiving demands exceed available emotional, social, or material resources. Chronic parental stress is associated with emotional exhaustion, depressive symptoms, sleep disturbances, impaired self-care, decreased treatment adherence, and increased risk of physical and psychological morbidity [4,5]. Among children, high parental stress is linked to maladaptive behavior, delayed development, and poorer psychosocial outcomes [5].

The Parenting Stress Index-Fourth Edition (PSI-4) is one of the most widely used instruments for evaluating parental stress, distinguishing between child-related factors (Child Domain) and caregiver-related factors (Parent Domain) [6]. Elevated percentile scores in subscales such as Health, Depression, Role Restriction, and Spouse/Partner Relationship reflect clinically significant stress. Despite its broad application across pediatric populations, few studies have investigated parental stress among mothers of children with total blindness, a condition associated with unique developmental and psychosocial challenges not observed in partial visual impairment.

Understanding children’s functional outcomes is equally important. The Children’s Visual Function Questionnaire (CVFQ) [7] and its Brazilian versions, QFVI-3 and QFVI-7 [8], assess functional vision-related quality of life across domains such as general vision concerns, functional competence, behavior, family impact, and treatment burden. Lower scores indicate greater impairment and have been associated with increased stress and decreased well-being within families of visually impaired children [7].

However, the simultaneous assessment of parental stress (PSI-4) and children’s vision-related functional quality of life (QFVI) in cases of total blindness remains a major gap in the literature. No Brazilian study to date has examined these constructs together, limiting the development of evidence-based interventions and public health strategies targeted to this population [9]. Considering the interplay between maternal psychological burden, child functional limitations, and socioeconomic determinants, research focusing on families of children with complete blindness is essential.

Total congenital blindness represents a distinct developmental and psychosocial condition compared with low vision. Unlike children with residual visual function, children with total blindness lack visual cues that support early autonomy, spatial orientation, and non-verbal social communication. This absence of visual input may increase caregiving demands, intensify reliance on parental mediation, and amplify the emotional and practical burden experienced by caregivers. Consequently, families of children with total blindness may face unique challenges related to daily routines, access to educational adaptations, and long-term developmental planning, which are not fully captured in studies that aggregate total blindness and low vision into a single category.

From a conceptual perspective, parental stress in the context of childhood total blindness can be understood as a multifactorial phenomenon arising from the interaction between child-related functional demands, maternal health and emotional well-being, and broader social and environmental contexts. Functional limitations associated with total blindness may increase caregiving workload and dependence, while maternal physical and emotional health can shape caregivers’ capacity to cope with these demands. Socioeconomic vulnerability, access to educational resources, and participation in work or leisure activities may further modulate these relationships, influencing both parental stress and children’s functional vision–related quality of life.

Based on this conceptual framework, the present study tested the following hypotheses: (H1) higher levels of parental stress would be associated with poorer maternal health indicators and higher emotional and somatic symptom burden; (H2) greater parental stress and maternal health vulnerability would be associated with lower children’s functional vision–related quality of life scores; (H3) socioeconomic disadvantage and limited access to educational adaptations would be associated with higher parental stress levels; and (H4) engagement in structured activities, such as maternal physical activity and school attendance, would be associated with more favorable functional vision-related quality of life outcomes in age-specific child subgroups.

Therefore, this study aims to evaluate parenting stress among mothers of children with total congenital blindness and to examine its associations with maternal health symptoms, socioeconomic conditions, and children’s functional vision-related quality of life measured through QFVI-3 and QFVI-7. By integrating standardized psychological and functional measures, this study seeks to fill a critical gap in pediatric ophthalmology and contribute to the development of family-centered clinical and psychosocial interventions.

## 2. Materials and Methods

### 2.1. Study Design

This was a cross-sectional, observational, and analytical study designed to evaluate parental stress, maternal health symptoms, and children’s functional vision-related quality of life in cases of total congenital blindness. The study followed the STROBE (Strengthening the Reporting of Observational Studies in Epidemiology) recommendations for observational research reporting [10] and complied with the ethical principles of the Declaration of Helsinki [11]. Additional methodological details are provided in Appendix A (Table A1).

### 2.2. Setting

Data collection took place in four public ophthalmology and rehabilitation centers in Goiania, Brazil. These centers provide specialized care for children with severe visual impairment, including pediatric ophthalmology, early stimulation, low-vision rehabilitation, orientation and mobility training, and multidisciplinary support.

### 2.3. Participants

#### 2.3.1. Inclusion Criteria

Eligible participants were biological mothers who were the primary caregivers of a child with total bilateral congenital blindness (no light perception). Additional criteria included having a child aged 0 to 12 years for PSI-4 analyses or 0 to 7 years for QFVI applicability, being literate in Portuguese (self-reported or interviewer-assisted), and providing written informed consent.

#### 2.3.2. Exclusion Criteria

Mothers were excluded if the child had additional neurological or systemic disabilities that could interfere with PSI-4 or QFVI responses, if the mother had untreated psychiatric conditions that impaired participation, or if the child’s blindness was acquired rather than congenital.

#### 2.3.3. Sampling Strategy

Because total congenital blindness is a rare condition, a consecutive convenience sampling strategy was used, following approaches commonly adopted in similar populations [12].

The total analytic sample consisted of 81 mother–child dyads, including children aged 0 to 12 years, which corresponds to the validated age range for the Parenting Stress Index–Fourth Edition (PSI-4). Analyses involving children’s functional vision-related quality of life were conducted in age-specific subsamples, restricted to children aged 0–3 years for the QFVI-3 and 3–7 years for the QFVI-7, in accordance with the validation criteria of each instrument. Children older than 7 years were therefore included only in analyses involving parental stress and maternal health variables.

### 2.4. Measures

#### 2.4.1. Parenting Stress Index, Fourth Edition, Full Form (PSI-4-FF)

Parental stress was assessed using the PSI-4-FF, a 120-item standardized instrument with excellent internal consistency (Cronbach alpha approximately 0.98) [6]. The PSI-4-FF includes:

- Child Domain (CD): Distractibility/Hyperactivity, Adaptability, Reinforces Parent, Demandingness, Mood, Acceptability.
- Parent Domain (PD): Competence, Isolation, Attachment, Role Restriction, De-pression, Spouse or Partner Relationship, Health.
- Life Stress Scale: 19 items assessing stressful events in the previous 12 months.

Scores were converted to standardized percentiles according to the instrument manual. Percentiles of 85 or above were classified as clinically elevated stress. The Defensive Responding Index was applied to evaluate response reliability.

#### 2.4.2. Quality of Functional Vision Questionnaire (QFVI-3 and QFVI-7)

Children’s functional vision and vision-related quality of life were evaluated using the Brazilian adaptations of QFVI-3 (ages 0 to 3 years) and QFVI-7 (ages 3 to 7 years), both derived from the Children’s Visual Function Questionnaire (CVFQ) [7,8].

These instruments assess six domains: general health, general vision, competence, personality, family impact, and treatment burden. Scores range from 0 (worst quality of life) to 100 (best). Non-applicable items were excluded from domain and total score calculations, following standard scoring procedures.

#### 2.4.3. Sociodemographic and Clinical Questionnaire

A structured questionnaire collected information on maternal age, education, marital status, occupation, and income. It also included psychosomatic symptoms such as fatigue, insomnia, headaches, and body pain, based on DSM-5 descriptors [13]. Additional variables included child school attendance, availability of educational adaptations, and access to rehabilitation and healthcare services. These variables were included as covariates in the analyses.

### 2.5. Procedures

Participants were recruited consecutively during routine appointments at the participating centers. Questionnaires were self-administered or completed with interviewer assistance when required. A pilot test with 10 mothers confirmed clarity and feasibility. Each assessment lasted approximately 45 to 60 min.

### 2.6. Bias Control

Several procedures were implemented to reduce bias. These included standardized administration protocols, interviewer training, verification of incomplete answers, and the use of validated psychometric instruments tailored to the Brazilian population. Interviewer assistance was provided only when needed to avoid misinterpretation while minimizing influence on responses.

### 2.7. Statistical Analysis

Statistical analyses followed the methodological standards recommended by the STROBE guidelines for observational studies [10]. Descriptive statistics summarized the characteristics of the sample. Categorical variables were expressed as frequencies and percentages. Continuous variables were described using means, medians, standard deviations, ranges, and 95 percent confidence intervals.

Normality of continuous variables was assessed using the Shapiro–Wilk test [14] and visual inspection of histograms and boxplots. Because most distributions did not meet normality assumptions, non-parametric methods were applied.

Group differences were examined using the Mann–Whitney U test and the Kruskal–Wallis test [15], with post hoc corrections for multiple comparisons when appropriate [16].

Associations between PSI-4 domains, Life Stress scores, QFVI-3 and QFVI-7 scores, and sociodemographic or clinical variables were evaluated using Spearman rank correlation coefficients [16]. Effect sizes were reported when applicable.

A two-tailed significance level of *p* < 0.05 was adopted. All analyses were performed using R statistical software, version 4.3 [17].

When multiple group comparisons were performed within the same domain, post hoc corrections for multiple testing were applied to control for type I error. Given the exploratory nature of the analyses and the relatively small sample size, conservative adjustments were adopted when appropriate, prioritizing transparency and interpretability of statistically significant findings.

### 2.8. Ethical Approval

The study was approved by the Research Ethics Committee of the Federal University of Goiás (CAAE 90833718.2.0000.5078; approval number 2.746.572). All mothers signed informed consent forms prior to participation. The study complied with Brazilian National Health Council Resolution 466/12 and with the Declaration of Helsinki [11].

## 3. Results

### 3.1. Child Characteristics

A total of 81 children with total congenital blindness participated in the study. The mean age was 6.07 ± 3.60 years (range: 0–12 years). The sample included 52 boys (64.2%) and 29 girls (35.8%).

Among the 58 children who were enrolled in school (71.6%), the majority attended regular schools (*n* = 50; 86.2% of students, or 61.7% of the total sample), whereas a smaller proportion were enrolled in specialized institutions for visually impaired students (*n* = 8; 9.9%).

Regarding educational support, most participants (64.2%) reported that their school offered no specific adaptations. Among those who did receive accommodations, the most common resources included the presence of an educational assistant (23.5%), the use of computer-based tools (14.8%), and accessibility adaptations such as enlarged print or tactile materials (13.6%). Because these items allow multiple responses, percentages are not mutually exclusive.

Participation in physical activity was reported by 26 children (32.1%), whereas 55 (67.9%) did not engage in structured exercise. Leisure activities were present in 36 children (44.4%), while 45 (55.6%) reported no regular leisure involvement.

**Figure 1:**
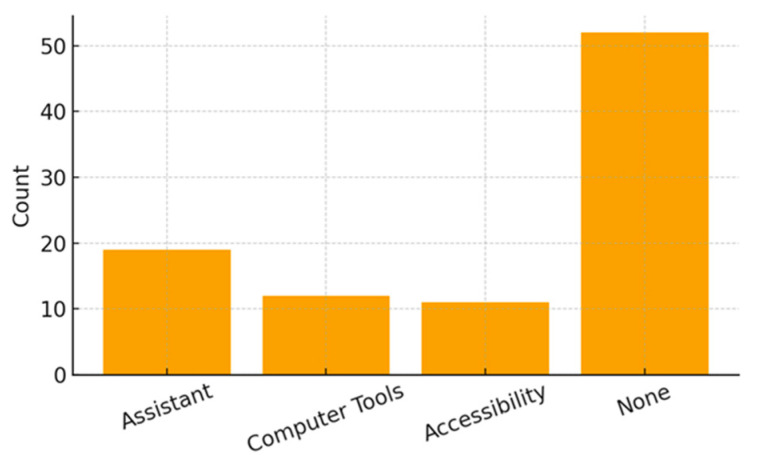
Distribution of Educational Resources.

**Figure 2:**
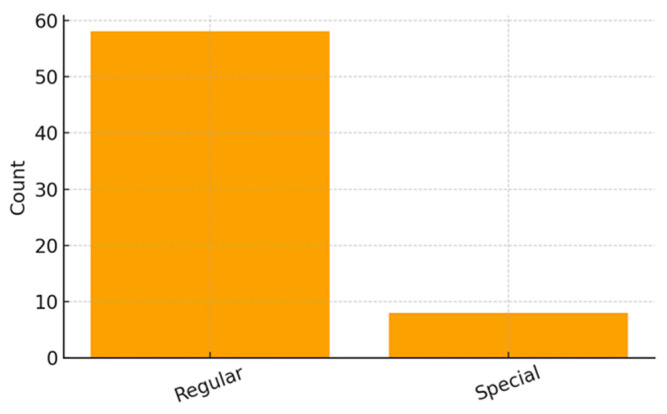
School Type Distribution.

**Figure 3:**
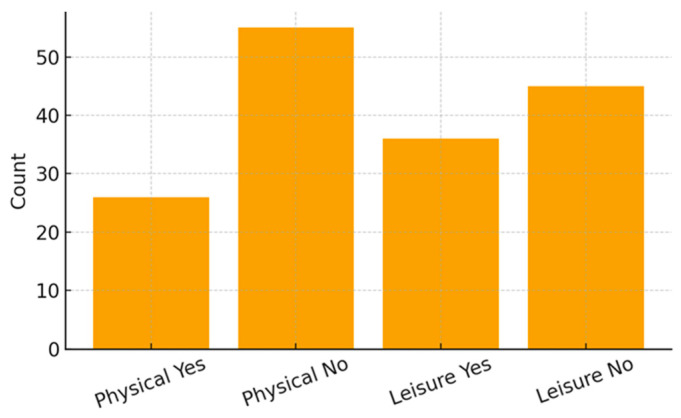
Physical Activity and Leisure Participation.

Figure 1, Figure 2 and Figure 3 illustrate the distribution of educational resources, school enrollment type, and participation in physical and leisure activities. Table 1 summarizes all sociodemographic and clinical characteristics of the participating children.

### 3.2. Family and Socioeconomic Characteristics

Family and socioeconomic data were obtained from all 81 mothers participating in the study. Most families belonged to lower socioeconomic strata, with 40.74% classified as class D according to ABEP criteria, 22.22% as class C2, 12.35% as class B2/C1, and 3.70% as class E, while 20.99% did not report income.

Maternal marital status also reflected a socioeconomic profile of high vulnerability: 58.02% of mothers were married, 18.52% were in a stable union, 11.11% were single, 7.41% divorced, and 4.94% widowed. Regarding employment, the majority of mothers (79.01%) did not work outside the home, and more than half of the families (54.32%) received social benefits. Only 29.63% of mothers reported having private health insurance.

In terms of family structure, 59 children (72.84%) lived with their father, whereas 21 (25.93%) did not; one mother did not provide this information (1.23%).

**Figure 4:**
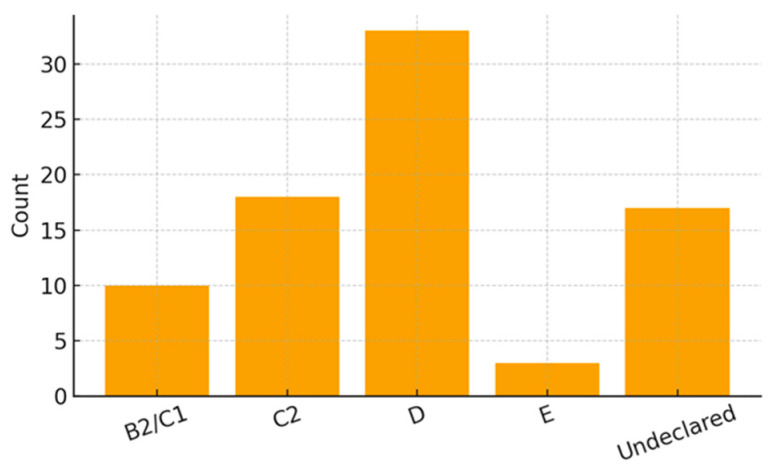
Socioeconomic Class Distribution (ABEP).

**Figure 5:**
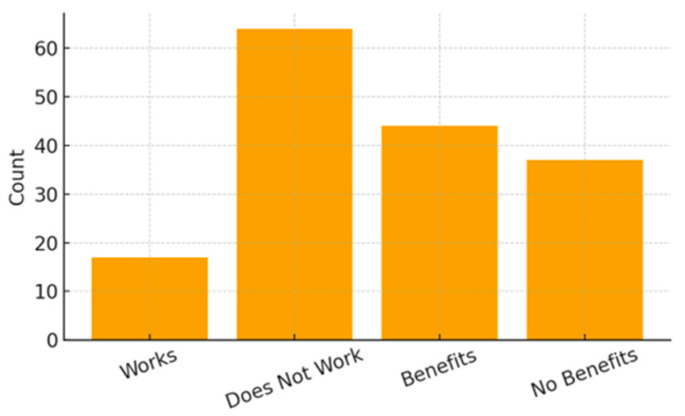
Maternal Employment and Social Benefits.

**Figure 6:**
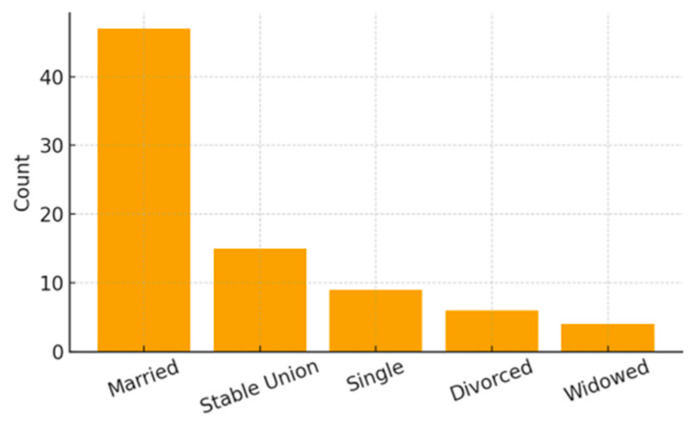
Maternal Marital Status Distribution.

Figure 4, Figure 5 and Figure 6 present the distribution of socioeconomic class, maternal employment and receipt of social benefits, and maternal marital status. Table 2 summarizes all family and socioeconomic characteristics of the sample.

### 3.3. Maternal Characteristics

Maternal characteristics were collected from all 81 participating mothers. The mean maternal age was 36.90 ± 7.89 years (range: 25–58 years). The mean body mass index (BMI) was 26.98 ± 4.76 kg/m^2^ (range: 18.73–39.54). Among mothers who were employed, the mean weekly workload was 37.00 ± 4.54 h.

Clinical indicators revealed hypertension in 16.05% of mothers and elevated blood glucose levels in 9.88%. Somatic symptoms were also highly frequent, including headache (62.96%), dizziness (25.93%), nausea (18.52%), and photophobia (40.74%). Emotional and psychological symptoms were similarly prominent, with high frequencies of sadness (70.37%), forgetfulness (76.54%), fatigue (82.72%), and insomnia (50.62%).

**Figure 7:**
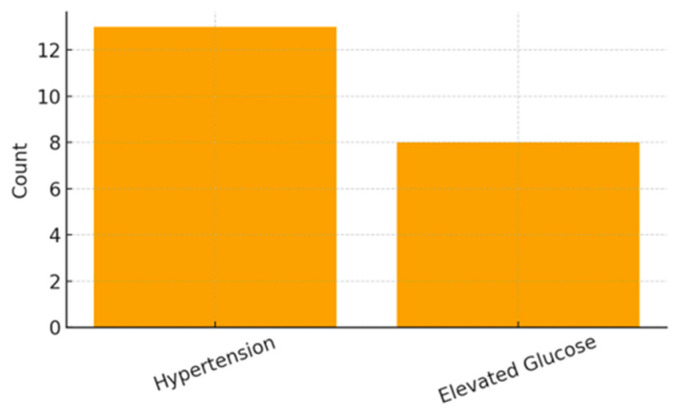
Maternal Clinical Conditions.

**Figure 8:**
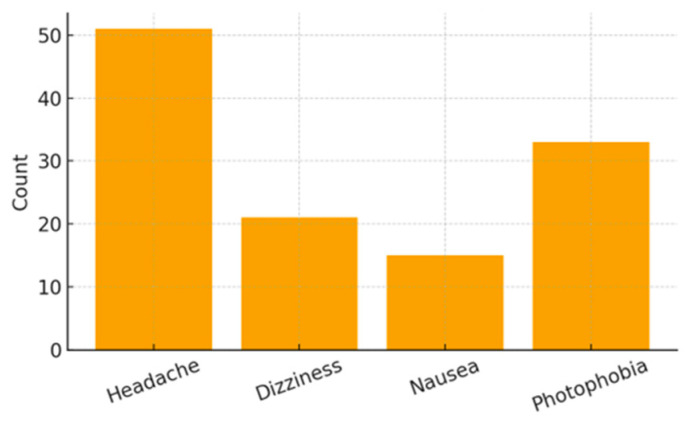
Maternal Somatic Symptoms.

**Figure 9:**
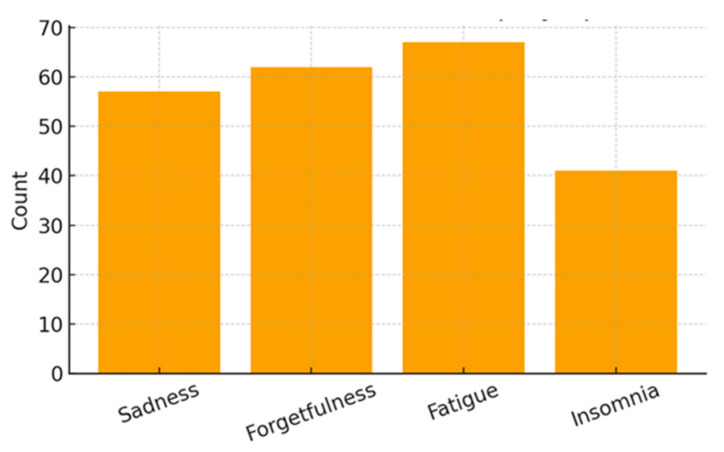
Maternal Emotional and Sleep Symptoms.

Figure 7, Figure 8 and Figure 9 present the distribution of clinical, somatic, and emotional characteristics among the mothers. Table 3 summarizes all maternal clinical, somatic, and emotional characteristics.

### 3.4. PSI-4 Child Domain Scores

#### 3.4.1. Subscale Percentile Distribution—PSI-4 Child Domain

The PSI-4 Child Domain comprises seven subscales that assess behavioral, emotional, and adaptive characteristics of the child as perceived by the caregiver. These subscales-Distractibility/Hyperactivity, Reinforces Parent, Mood, Acceptability, Adaptability, Demandingness, and the Total Child Domain score-form the core structure of the instrument. Percentile values reported here follow standardized scoring procedures based on the PSI-4 international normative dataset. This subsection presents descriptive statistics for each subscale, including mean percentile, standard deviation, and 95% confidence intervals.

The subscales demonstrated notable variation in mean percentile values. Higher means were observed for Adaptability (71.62), Demandingness (69.17), and Distractibility/Hyperactivity (68.47). Intermediate means appeared in Acceptability (67.80) and the Total Child Domain score (66.70). In contrast, Reinforces Parent (49.89) and Mood (55.84) displayed comparatively lower mean percentiles. These findings offer a descriptive summary of the relative distribution of scores among the seven subscales.

Standard deviations ranged from approximately 21 to 27 percentile points, indicating moderate variability within the sample. Confidence interval widths also varied, reflecting heterogeneous dispersion across subscales: narrower intervals denote greater score concentration, whereas wider intervals suggest broader variability. Together, these descriptive measures illustrate the distributional characteristics of the PSI-4 Child Domain within this cohort.

**Figure 10:**
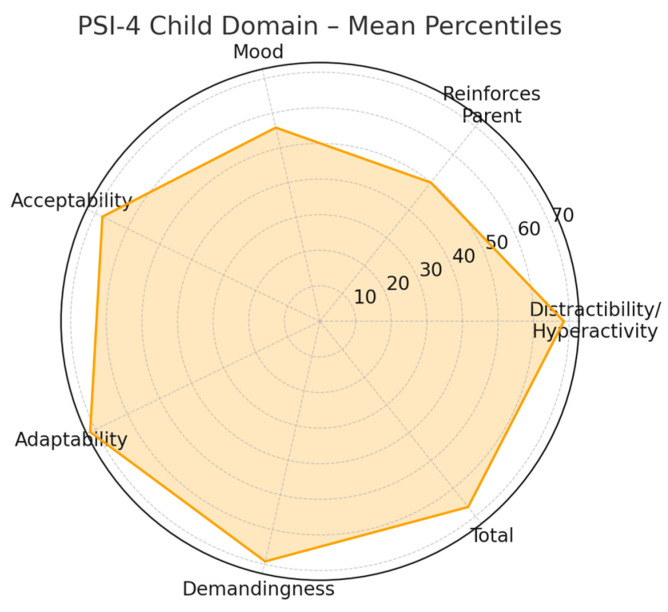
Radar Chart PSI-4 Child Domain.

Figure 10 provides a radar chart representation of the mean percentiles across the seven PSI-4 Child Domain subscales. This visualization enables immediate recognition of differences in subscale magnitudes: subscales with higher mean values extend farther outward, whereas those with lower means remain closer to the center. The figure serves as an objective graphical summary without offering inferential interpretation. A detailed numerical distribution of these percentile scores is presented in Table 4.

#### 3.4.2. Group Comparisons for Child Domain

Non-parametric group comparisons using Mann–Whitney U and Kruskal–Wallis tests were conducted to evaluate differences in PSI-4 Child Domain percentile scores across clinical, sociodemographic, and maternal health factors. Independent variables examined included ophthalmologic diagnosis, child age, parental education, socioeconomic class (ABEP), time since diagnosis, family composition (living with the father or not), and several maternal symptoms such as headache, intolerance to light or noise, sadness, fatigue, body pain, insomnia, difficulty falling asleep, and early morning awakenings.

The statistical report indicated that child age, time since diagnosis, parental education and socioeconomic class did not show significant group differences. However, multiple maternal symptoms and family structure variables were significantly associated with higher Child Domain percentile scores. Children who did not live with their father exhibited higher scores (*p* = 0.039). Elevated percentile scores were also observed among children whose mothers reported headache (*p* = 0.003), intolerance to light or noise (*p* = 0.003), sadness or emotional distress (*p* = 0.036), fatigue (*p* = 0.011), body pain (*p* = 0.037), insomnia (*p* = 0.023), difficulty falling asleep (*p* = 0.038), and early morning awakenings (*p* = 0.013).

**Figure 11:**
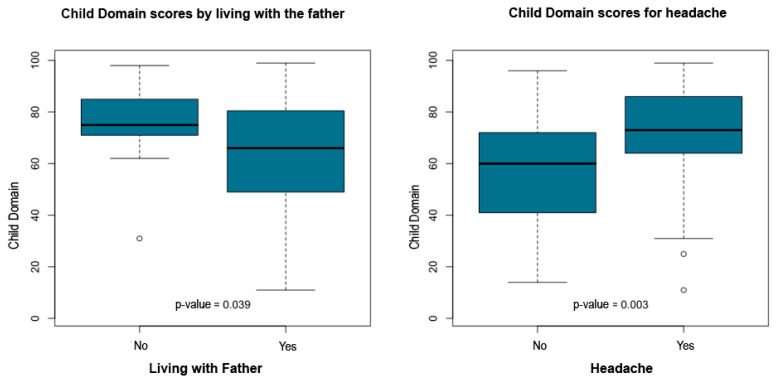
PSI-4 Child Domain scores by living with the father and for headache (boxplot).

**Figure 12:**
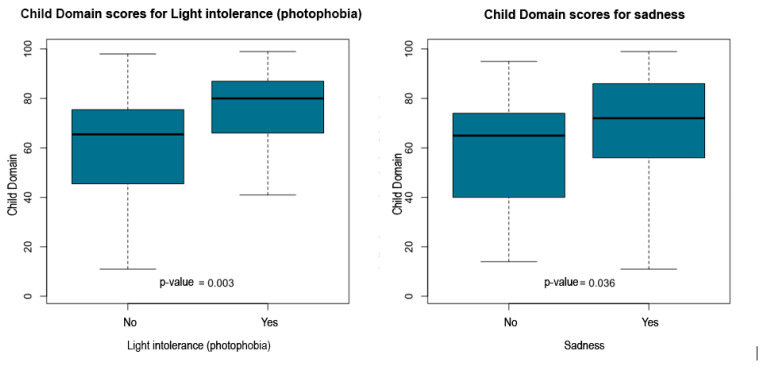
PSI-4 Child Domain scores by maternal light intolerance and sadness (boxplots).

**Figure 13:**
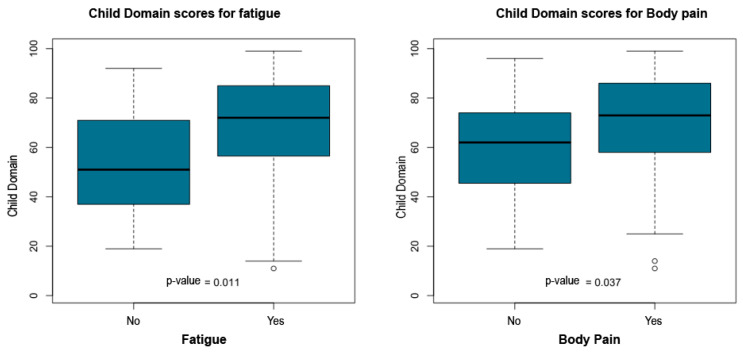
PSI-4 Child Domain scores by maternal fatigue and body pain (boxplots).

**Figure 14:**
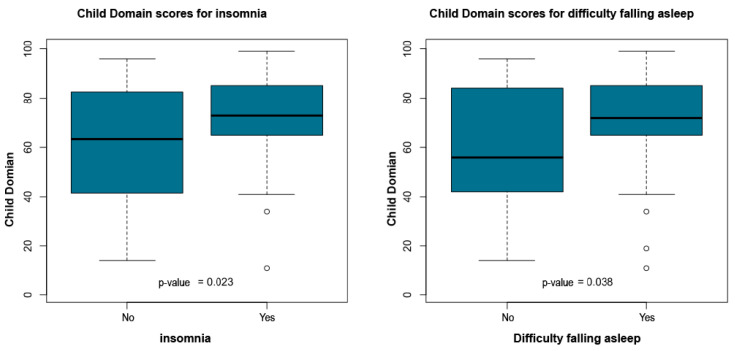
PSI-4 Child Domain scores by insomnia and difficulty falling asleep (boxplot).

**Figure 15:**
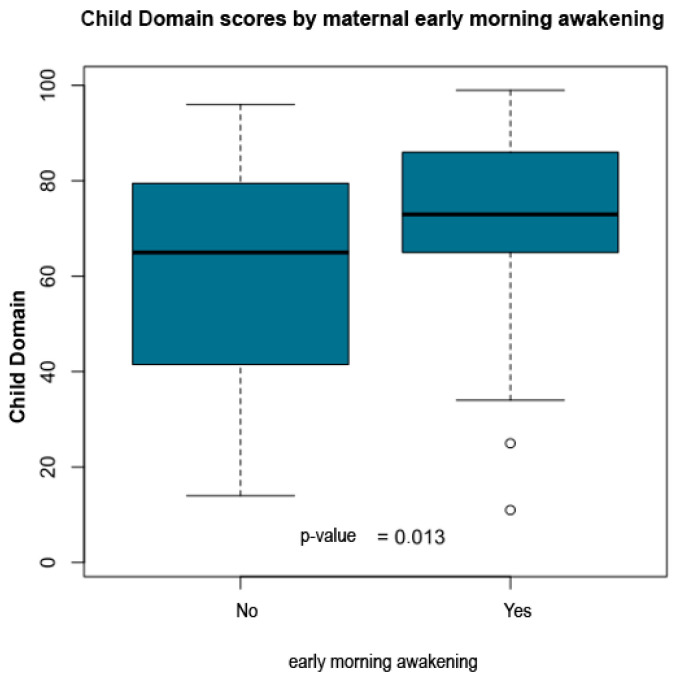
PSI-4 Child Domain scores by maternal early morning awakening (boxplot).

Table 5 summarizes the significant associations between PSI-4 Child Domain scores and maternal or family variables. Figure 11, Figure 12, Figure 13, Figure 14 and Figure 15 present boxplot visualizations of the variables showing statistically significant group differences, using the total Child Domain percentile score as the dependent measure.

### 3.5. PSI-4 Parent Domain Scores

#### 3.5.1. Subscale Percentile Distribution PSI-4 Parent Domain

The PSI-4 Parent Domain is composed of eight subscales that evaluate perceived parental functioning across emotional, physical, relational, and role-related dimensions. These subscales include: Competence, Isolation, Attachment, Health, Role Restriction, Depression, Spouse/Partner Relationship, and the Total Parent Domain score. Percentile values used in this analysis were derived from the standardized scoring procedures of the PSI-4 normative system.

Mean percentile scores varied across the eight subscales. Higher mean values were observed for Health (74.31), Depression (73.02), Competence (66.46), Role Restriction (66.54), and the Total Parent Domain score (66.09). Moderate values were found for Isolation (65.89) and Attachment (59.07), whereas the lowest percentile was observed for Spouse/Partner Relationship (47.59). These descriptive values summarize the distributional profile of the parent-related stress indicators.

Standard deviations ranged from approximately 22 to 31 percentile points, indicating variable dispersion across subscales. Confidence intervals also differed, with narrower intervals showing greater clustering of participant scores and wider intervals reflecting higher heterogeneity. Together, these indicators provide a distributional overview of variability within the Parent Domain.

**Figure 16:**
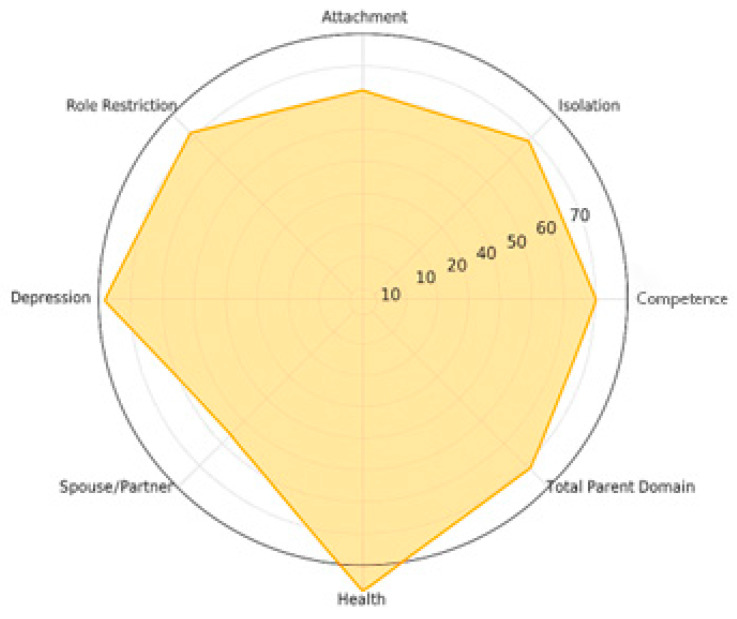
Radar Chart PSI-4 Parent Domain.

Figure 16 displays a radar chart summarizing the mean percentile scores for the eight Parent Domain subscales, as detailed in Table 6. The visualization allows for immediate inspection of the relative magnitude of each subscale while maintaining an objective, non-interpretative representation.

#### 3.5.2. Group Comparisons—PSI-4 Parent Domain

Non-parametric group comparisons (Mann–Whitney U and Kruskal–Wallis tests) were performed to examine differences in PSI-4 Parent Domain percentile scores across clinical, socioeconomic, and maternal health variables. Independent variables included family income (ABEP classification), parental education, maternal age, ophthalmologic diagnosis, time since diagnosis, family composition (living with the father), and multiple maternal symptoms.

Family income, parental education, maternal age, and ophthalmologic diagnosis did not produce statistically significant group differences in Parent Domain scores.

In contrast, several maternal symptoms and one family structure variable demonstrated statistically significant associations with higher Parent Domain percentile scores. Mothers reporting sadness (*p* < 0.001), fatigue (*p* = 0.027), body pain (*p* = 0.001), insomnia (*p* = 0.001), difficulty falling asleep (*p* = 0.001), or early morning awakenings (*p* = 0.040) exhibited higher Parent Domain scores. Additionally, intolerance to light or noise showed a strong association with elevated stress levels (*p* < 0.001). Children who did not live with their father also presented higher Parent Domain scores (*p* = 0.015).

Table 7 summarizes all significant associations between Parent Domain scores and maternal or family variables.

### 3.6. Quality of Family Vision Impact (QFVI)

The Quality of Family Vision Impact (QFVI) instrument was used to assess the functional, emotional, and social effects of pediatric visual impairment on family dynamics. Two age-specific versions were applied to ensure developmental appropriateness: QFVI-3 for children aged 0–3 years and QFVI-7 for children aged 3–7 years. Both instruments evaluate the extent to which the child’s visual condition influences family routines, parental well-being, and overall household functioning.

#### 3.6.1. QFVI 3 (0–3 Years)

The QFVI-3 was administered to caregivers of children aged 0–3 years. Its domains assess early-childhood–related aspects, including parental emotional concerns, perceived disruption of daily routines, functional limitations related to early development, and family-related functioning. Scores were converted to standardized percentages (0–100) according to instrument guidelines.

**Figure 17:**
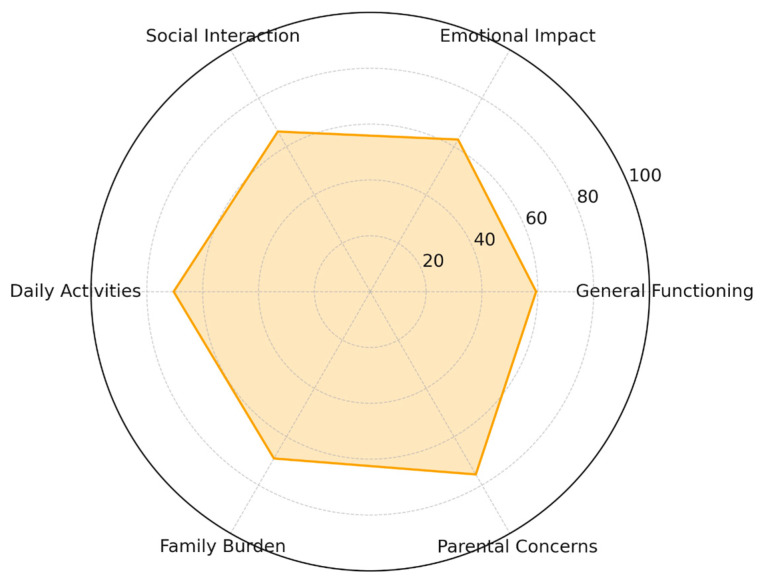
Radar Chart QFVI-3 (0–3 years).

Descriptive analysis showed moderate variability across domains, reflecting heterogeneity in how families experience the consequences of severe visual impairment or blindness during early developmental stages, as shown in Table 8. A radar-chart visualization of domain percentile profiles is shown in Figure 17.

#### 3.6.2. QFVI 7 (3–7 Years)

The QFVI-7 evaluates similar constructs but expands them to reflect preschool and early-school developmental demands, including autonomy, peer interaction, social participation, and school-related functioning. The domains maintain the same conceptual framework while incorporating age-appropriate indicators.

**Figure 18:**
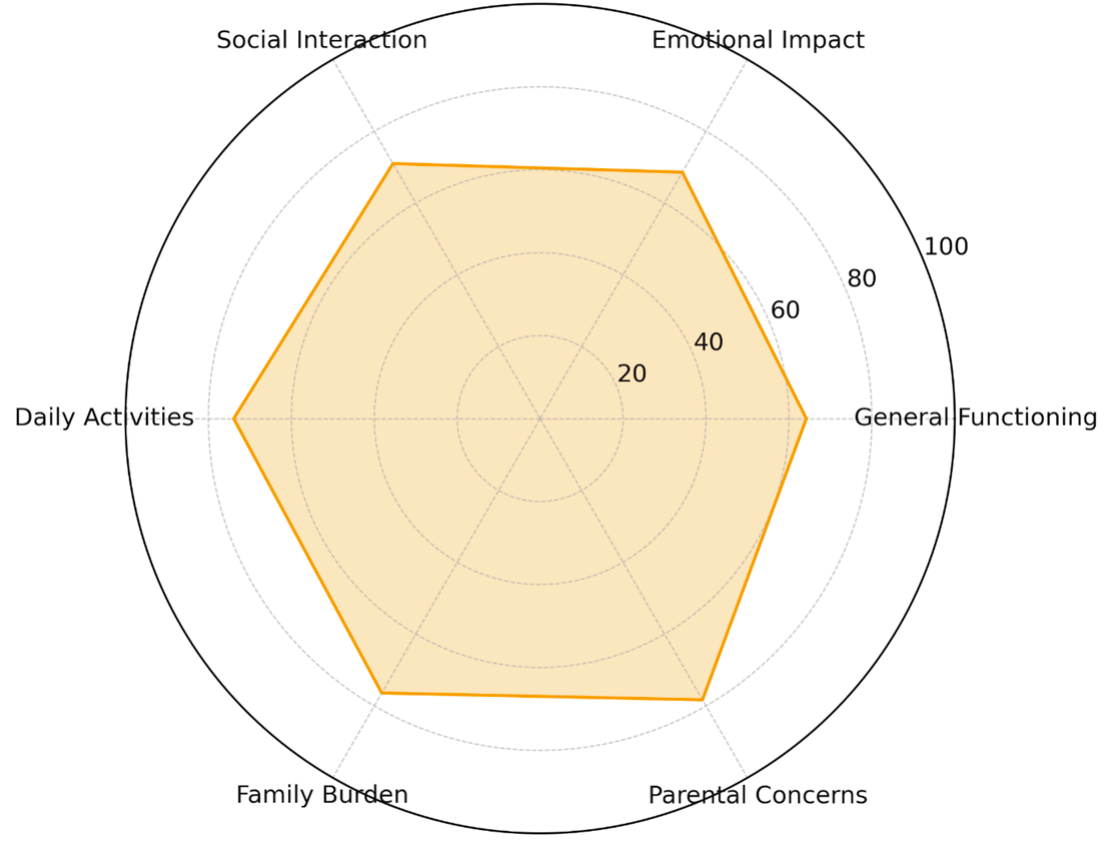
Radar Chart QFVI-7 (3–7 years).

Descriptive values indicated consistent emotional and functional impacts across families, with variability patterns similar to those observed in younger children, as detailed in Table 9. The multidomain profile for QFVI-7 is illustrated in Figure 18

#### 3.6.3. Group Comparisons for QFVI

Group comparisons were conducted to examine whether QFVI-3 and QFVI-7 scores varied according to socioeconomic, demographic, educational, and maternal health variables. Because the data did not meet normality assumptions, non-parametric tests (Mann–Whitney U and Kruskal–Wallis) were applied. Only variables with statistically significant differences (*p* < 0.05) are reported.

Significant findings for QFVI-3 (0–3 years) are summarized in Table 10, four variables demonstrated significant group differences in QFVI-3 scores:

1. Physical activity (*p* = 0.038): Mothers who engaged in regular physical activity had higher QFVI-3 scores compared with those who did not.
2. Nausea (*p* = 0.044): Mothers reporting nausea had lower QFVI-3 scores than those without nausea.
3. Fatigue (*p* = 0.038): Mothers reporting fatigue had lower QFVI-3 scores.
4. Body pain (*p* = 0.006): Mothers reporting body pain had lower QFVI-3 scores.

**Table 10:** Significant Group Comparisons for QFVI-3.

Variable	Groups Compared	N (G1/G2)	Mean (G1/G2)	*p*-Value	Interpretation
Physical activity	No vs. Yes	14/4	51.37/65.62	0.038	Higher QFVI-3 among mothers who exercise
Nausea	No vs. Yes	15/3	56.61/44.19	0.044	Lower QFVI-3 among mothers with nausea
Fatigue	No vs. Yes	4/14	64.58/51.67	0.038	Lower QFVI-3 among mothers with fatigue
Body pain	No vs. Yes	6/12	63.65/49.98	0.006	Lower QFVI-3 among mothers with body pain

G1 = Group 1; G2 = Group 2.

Significant findings for QFVI-7 (3–7 years) are summarized in Table 11; four variables demonstrated significant group differences in QFVI-7 scores:

5. Child attends school (*p* = 0.003): Caregivers of children who attended school had higher QFVI-7 scores compared with those whose children did not attend school.
6. Nausea (*p* = 0.026): Mothers reporting nausea had lower QFVI-7 scores than those without nausea.
7. Light intolerance (*p* = 0.034): Caregivers reporting light intolerance had lower QFVI-7 scores.
8. Difficulty falling asleep (*p* = 0.002): Mothers reporting difficulty falling asleep had lower QFVI-7 scores.

**Table 11:** Significant Group Comparisons for QFVI-7.

Variable	Groups Compared	N (G1/G2)	Mean (G1/G2)	*p*-Value	Interpretation
Child attends school	No vs. Yes	6/28	41.87/54.12	0.003	Higher QFVI-7 when the child attends school
Nausea	No vs. Yes	25/9	53.99/46.32	0.026	Lower QFVI-7 among mothers with nausea
Light intolerance	No vs. Yes	20/14	54.86/47.82	0.034	Lower QFVI-7 among mothers with light intolerance
Difficulty falling asleep	No vs. Yes	19/15	56.01/46.83	0.002	Lower QFVI-7 among mothers with difficulty falling asleep

G1 = Group 1; G2 = Group 2.

### 3.7. Bivariate Associations Between PSI-4, Life Stress, and QFVI

Spearman’s rank correlations were performed to examine associations among PSI-4 domains, life stress indicators, and family impact scores measured by QFVI-3 and QFVI-7. A strong and statistically significant correlation was observed between the PSI-4 Child Domain and the PSI-4 Parent Domain (ρ = 0.59, *p* < 0.001), indicating concordance between stress related to child characteristics and stress related to parental functioning. No additional significant correlations were found between PSI-4 domains and other variables.

Associations between PSI-4 and life stress indicators were also evaluated. Neither the Child Domain nor the Parent Domain demonstrated significant correlations with the Life Stress indicator (all *p* > 0.05). However, Life Stress Score and Life Stress Percentile were strongly correlated with each other (ρ = 0.94, *p* < 0.001), reflecting internal consistency within the life stress construct.

Correlations between PSI-4 domains and family impact (QFVI-3 and QFVI-7) were examined next. No significant correlations were observed between PSI-4 Child or Parent domains and QFVI-3 or QFVI-7 (all *p* > 0.30). In contrast, age-specific patterns emerged when evaluating Life Stress and QFVI. For families of younger children (QFVI-3), no significant associations were found. Among families of children aged 3–7 years, Life Stress Score (ρ = −0.28, *p* = 0.013) and Life Stress Percentile (ρ = −0.26, *p* = 0.017) showed significant negative correlations with QFVI-7, indicating greater family impact in the presence of higher contextual stress.

### 3.8. Summary of Exploratory Modeling Results

Exploratory regression models were conducted to examine associations between child- and caregiver-related variables and overall parental stress (PSI-4 Total Stress percentile). These analyses were intended to generate hypotheses rather than to establish causal relationships and should therefore be interpreted with caution. Variables entered into the models were selected based on theoretical relevance, prior empirical evidence, and statistically significant associations observed in univariate analyses. Given the sample size constraints inherent to rare conditions, the number of predictors included in multivariable models was intentionally limited to reduce the risk of overfitting. Within this exploratory framework, higher PSI-4 Total Stress percentiles were positively associated with maternal depressive symptom scores, lower socioeconomic class, and not living with the child’s father, while total QFVI scores showed a negative association with parental stress levels.

Variables that did not retain statistical significance after adjustment included maternal age, child age, and regular physical activity. These findings indicate that only a subset of demographic and clinical variables remained statistically significant in the adjusted model.

A summary of the regression coefficients and statistical outputs is presented in Table 12, which provides an overview of the variables included, parameter estimates, and model fit indices.

Model fit was evaluated using adjusted R^2^ values, which indicated modest explanatory power consistent with exploratory modeling in small samples. Multicollinearity diagnostics were assessed using variance inflation factors (VIF), with all values remaining below commonly accepted thresholds, indicating no evidence of problematic multicollinearity among predictors.

### 3.9. Summary of Key Quantitative Results (Descriptive Summary)

Across all analyses, the PSI-4 Parent Domain showed higher percentile scores than the Child Domain, with the highest values observed in the Depression and Health subscales. Within the Child Domain, Adaptability and Demandingness presented the highest mean percentiles.

For visual functioning, both QFVI-3 and QFVI-7 demonstrated lower scores in domains related to general functioning and family impact, indicating greater reported challenges in these areas compared with other QFVI domains.

In the bivariate analyses, the only strong internal association within PSI-4 was the positive correlation between the Child and Parent Domains. No significant correlations were found between PSI-4 domains and Life Stress or between PSI-4 domains and QFVI-3/QFVI-7. Among families of children aged 3–7 years, Life Stress indicators showed significant negative correlations with QFVI-7, whereas no such associations were observed for QFVI-3.

Exploratory regression models identified maternal depressive symptoms, lower socioeconomic class, not living with the child’s father, and lower QFVI total scores as variables that remained significantly associated with higher PSI-4 Total Stress percentiles, while maternal age, child age, and physical activity were not significant after adjustment.

## 4. Discussion

This study evaluated parental stress, maternal mental health, and vision-related quality of life in families of children with total congenital blindness. Overall, the findings demonstrated elevated parental burden across several dimensions of the PSI-4, particularly within the Parent Domain, where the highest percentile scores were observed in the Depression and Health subscales. These domains reflect somatic symptoms, emotional exhaustion, and decreased perceived competence-core components of caregiver overload described in the PSI-4 manual [6]. Although most mothers remained within normative stress levels, approximately 21% fell into the clinically significant or pathological range, aligning with previous studies documenting substantial psychological demands among caregivers of children with sensory disabilities [18].

In contrast to profiles commonly observed in neurological or genetic disorders, children in this study did not display globally maladaptive behavioral patterns. PSI-4 Child Domain scores indicated elevated demands primarily in Adaptability, Demandingness, and Distractibility/Hyperactivity—findings consistent with literature describing attentional and regulatory challenges in blind children [19]. At the same time, preserved scores in Reinforces Parent and Humor suggest strong affective bonding, echoing prior research reporting robust emotional reciprocity between blind children and caregivers [20]. Taken together, the findings indicate that parental stress in the context of total childhood blindness is more strongly associated with maternal psychological vulnerability, socioeconomic hardship, and environmental constraints than with child behavioral characteristics alone. This pattern underscores the relevance of caregiver-related and contextual factors in shaping family experiences of severe visual impairment.

Quality-of-life findings from QFVI-3 and QFVI-7 showed moderate impairments in domains related to general functioning, daily activities, family burden, and parental concerns-dimensions previously highlighted in the original CVFQ/QFVI studies by Felius et al. [7] and supported by Brazilian validation data [8]. Across both age groups, the most consistent predictors of lower QFVI scores were maternal physical symptoms, including nausea, fatigue, pain, and sleep disturbances. This pattern reinforces a reciprocal relationship between caregiver well-being and the perceived impact of childhood blindness on family functioning. For QFVI-7, school attendance was associated with better functional and emotional outcomes, consistent with evidence that structured educational environments and peer interaction are linked to improved adaptation in visually impaired children [12,21]. Among younger children, maternal physical activity was associated with higher QFVI-3 scores, in line with broader evidence that exercise mitigates stress and depressive symptoms in caregivers [13,22].

A central finding of this study is that maternal psychological health, socioeconomic vulnerability, and family structure were more strongly associated with parental stress than the child’s functional profile alone. Maternal symptoms consistent with depressive syndromes-including insomnia, sadness, low energy, headaches, and bodily pain-were strongly associated with elevated Parent Domain scores, paralleling previous studies linking parental stress and depressive symptoms in families of blind children [18] and theoretical models suggesting that chronic parenting stress may evolve into clinically relevant depressive states [23]. Mothers not living with the child’s father also exhibited significantly higher stress, which is coherent with robust evidence on the protective role of partner support in families of children with disabilities [24].

An important contribution of this study lies in the integrated interpretation of parental stress (PSI-4), contextual stressors (Life Stress), and children’s functional vision–related quality of life (QFVI). Rather than operating independently, these dimensions appear to be interconnected within a shared family context. While PSI-4 domains captured caregivers’ emotional, physical, and relational burden, Life Stress indicators reflected broader environmental pressures, and QFVI scores indexed the perceived functional impact of blindness on daily family life. The observed associations—particularly between Life Stress and QFVI-7—suggest that contextual adversity may exacerbate functional challenges during early school years, even in the absence of direct associations between PSI-4 domains and QFVI scores.

When contrasted with studies that include children with partial visual impairment or low vision, the present findings suggest a potential gradient effect related to the severity of visual loss. Research in low-vision populations often reports milder functional restrictions and greater child autonomy, alongside lower caregiver stress levels compared with families of totally blind children. The absence of residual vision in total blindness may intensify dependence, caregiving workload, and environmental mediation, thereby amplifying maternal stress and family burden. This distinction reinforces the importance of analyzing total blindness as a separate clinical and psychosocial condition rather than aggregating it with low-vision phenotypes.

Socioeconomic vulnerability (ABEP classes C2-D-E) was likewise associated with higher PSI-4 scores, mirroring international literature that links financial strain to heightened caregiver stress, reduced access to rehabilitation services, and disruption of family routines [25]. Furthermore, the observed associations between Life Stress indicators and QFVI-7 underscore how contextual pressures-such as transportation difficulties, limited environmental accessibility, and inconsistent inclusion practices-can amplify both functional and psychosocial challenges, particularly during the preschool and early school years.

Despite these burdens, indicators of resilience were evident. The majority of mothers (approximately 79%) remained within normal stress ranges, and patterns of strong affective bonding and relatively low scores in Spouse/Partner Relationship stress are compatible with previous descriptions of family cohesion as a resilience-related characteristic in childhood blindness. Similar resilience profiles have been reported in pediatric oncology, where mothers of children with retinoblastoma exhibit substantial adaptive capacity despite intense treatment demands [25]. These converging findings suggest that many families develop meaningful coping strategies, even under conditions of chronic strain.

From a public health perspective, these findings support the implementation of family-centered and multidisciplinary care pathways within the Brazilian Unified Health System (SUS). Practical strategies include routine screening for parental stress and maternal depressive symptoms in pediatric ophthalmology and rehabilitation services; early referral to psychological or social support when elevated stress is identified; integration of orientation and mobility training with early stimulation programs; and close collaboration with the education sector to facilitate school inclusion, accessibility adaptations, and teacher training. Such coordinated approaches may mitigate caregiver burden and promote more favorable functional and psychosocial outcomes for children with total blindness.

Taken together, the results support a multifactorial explanatory model: although total childhood blindness imposes substantial functional and caregiving demands, maternal psychological vulnerability, socioeconomic hardship, and broader contextual stressors appear to play dominant roles in determining parental stress. Importantly, several modifiable variables were identified-such as maternal physical activity, school inclusion, and family cohesion-indicating tangible pathways for intervention. These findings strengthen the argument for structured, family-centered care, systematic screening for maternal depressive symptoms, clear referral pathways, multidisciplinary rehabilitation, and public policies that improve accessibility, transportation, and social inclusion for children with total blindness, in accordance with the ethical principles outlined in the PSI-4 Manual and the Declaration of Helsinki [11].

## 5. Conclusions

This study offers a comprehensive characterization of parental stress, maternal psychological functioning, and vision-related quality of life in families of children with total blindness, regardless of etiology. Mothers frequently reported depressive symptoms, somatic complaints, sleep disturbances, and reduced psychological energy. Although most remained within normative stress levels, approximately 21% fell within clinically significant or pathological ranges, indicating substantial emotional burden. At the same time, many families demonstrated resilience-based coping strategies supported by family cohesion and strong affective bonding.

Despite the severity of visual impairment, children did not exhibit globally maladaptive behavioral profiles. Instead, parental stress was more strongly associated with maternal emotional vulnerability, socioeconomic hardship, and environmental barriers than with child temperament or the underlying etiology of blindness. Vision-related quality-of-life scores showed moderate impairments across domains. Age-specific patterns emerged: among children aged 0–3 years, higher QFVI-3 scores were associated with maternal physical activity, whereas among children aged 3–7 years, school attendance was associated with higher QFVI scores. These findings highlight the importance of caregiver well-being, structured routines, and enriched environments in supporting developmental adaptation.

Although the PSI-4 Child and Parent Domains were not significantly associated with QFVI-3 or QFVI-7, Life Stress indicators demonstrated a significant negative correlation with QFVI-7. This suggests that contextual pressures—rather than parent–child relational stress alone—exert a stronger influence on family functioning during the preschool and early school years.

Taken together, these findings highlight the need for family-centered, multidisciplinary, and psychosocially informed clinical care for children with total blindness of any etiology. Integrating maternal mental-health screening, supportive psychological interventions, early vision rehabilitation, orientation and mobility training, and strategies to promote school inclusion is essential. Future longitudinal and interventional studies are needed to clarify causal pathways, evaluate targeted support strategies, and inform public policies aimed at reducing parental burden and improving both caregiver and child outcomes in the context of severe visual impairment.

## Figures and Tables

**Table 1:** Child Clinical and Sociodemographic Characteristics.

Variable	Value	Unit/Categories	Notes
Number of participants	81	*n*	
Age (mean ± SD)	6.07 ± 3.60	years	No median or range available
Sex—Male	52 (64.2%)	*n* (%)	
Sex—Female	29 (35.8%)	*n* (%)	
School Attendance—Yes	58 (71.6%)	*n* (%)	
School Attendance—No	23 (28.4%)	*n* (%)	
School Type—Regular	50 (61.7%) ^1^	*n* (%)	
School Type—Special	8 (9.9%)	*n* (%)	
Educational Resource: Assistant	19 (23.5%) ^2^	*n* (%)	
Educational Resource: Computer Tools	12 (14.8%)	*n* (%)	
Educational Resource: Accessibility Adaptations	11 (13.6%)	*n* (%)	
Educational Resource: None	52 (64.2%)	*n* (%)	
Physical Activity—Yes	26 (32.1%)	*n* (%)	
Physical Activity—No	55 (67.9%)	*n* (%)	
Leisure Activities—Yes	36 (44.4%)	*n* (%)	
Leisure Activities—No	45 (55.6%)	*n* (%)	

^1^ Percent calculated from N = 81; raw = 50/58 = 86.2%; ^2^ Multiple responses possible

**Table 2:** Family and Socioeconomic Characteristics.

Variable	Category/Value	*n* (%)	Notes
Socioeconomic Class (ABEP)	B2/C1	10 (12.35%)	
	C2	18 (22.22%)	
	D	33 (40.74%)	Majority
	E	3 (3.70%)	
	Undeclared	17 (20.99%)	
Maternal Employment	Does not work outside home	64 (79.01%)	
	Works outside home	17 (20.99%)	
Receives Social Benefits	Yes	44 (54.32%)	
	No	37 (45.68%)	
Health Insurance	No	57 (70.37%)	
	Yes	24 (29.63%)	
Maternal Marital Status	Married	47 (58.02%)	
	Stable union	15 (18.52%)	
	Single	9 (11.11%)	
	Divorced	6 (7.41%)	
	Widowed	4 (4.94%)	
Child Lives with Father	Yes	59 (72.84%)	
	No	21 (25.93%)	
	Missing ^1^	1 (1.23%)	

^1^ One participant did not respond to this item.

**Table 3:** Maternal Characteristics.

Variable	Category/Value	*n* (%)/Mean ± SD	Range/Notes
Maternal Age (years)	-	36.90 ± 7.89	25–58
BMI (kg/m^2^)	-	26.98 ± 4.76	18.73–39.54
Weekly Work Hours	-	37.00 ± 4.54	30–40 h
Hypertension	Yes	13 (16.05%)	-
	No	68 (83.95%)	-
Elevated Blood Glucose	Yes	8 (9.88%)	-
	No	73 (90.12%)	-
Headache	Yes	51 (62.96%)	No = 30 (37.04%)
Dizziness	Yes	21 (25.93%)	No = 60 (74.07%)
Nausea	Yes	15 (18.52%)	No = 66 (81.48%)
Photophobia	Yes	33 (40.74%)	No = 48 (59.26%)
Sadness	Yes	57 (70.37%)	No = 24 (29.63%)
Forgetfulness	Yes	62 (76.54%)	No = 19 (23.46%)
Fatigue	Yes	67 (82.72%)	No = 14 (17.28%)
Insomnia	Yes	41 (50.62%)	No = 40 (49.38%)

**Table 4:** PSI-4 Child Domain Percentile Scores.

Subscale	Mean Percentile	SD	95% CI (Lower)	95% CI (Upper)
Distractibility/Hyperactivity	68.47	23.30	63.46	73.58
Reinforces Parent	49.89	25.90	44.31	55.79
Mood	55.84	27.14	50.21	61.79
Acceptability	67.80	25.06	62.09	73.01
Adaptability	71.62	22.57	66.39	76.48
Demandingness	69.17	24.08	63.90	74.53
Total Child Domain	66.70	21.67	61.00	72.00

**Table 5:** Group Comparisons for PSI-4 Child Domain Scores.

Variable	Direction of Association	*p*-Value
Lives with father	Higher scores when NOT living with father	0.039
Maternal headache	Higher scores when present	0.003
Intolerance to light/noise	Higher scores when present	0.003
Maternal sadness/distress	Higher scores when present	0.036
Maternal fatigue	Higher scores when present	0.011
Maternal body pain	Higher scores when present	0.037
Maternal insomnia	Higher scores when present	0.023
Difficulty falling asleep	Higher scores when present	0.038
Early morning awakenings	Higher scores when present	0.013

**Table 6:** Parent Domain Percentile Scores.

Subscale	Mean Percentile	SD	95% CI (Lower)	95% CI (Upper)
Competence	66.46	23.90	61.40	71.03
Isolation	65.89	25.98	60.75	71.16
Attachment	59.07	22.81	54.12	64.16
Role Restriction	66.54	29.36	60.19	73.15
Depression	73.02	22.98	68.01	78.08
Spouse/Partner	47.59	31.31	41.16	54.54
Health	74.31	24.49	69.01	79.48
Total Parent Domain	66.09	24.03	60.95	71.22

**Table 7:** Group Comparisons for PSI-4 Parent Domain Scores.

Variable	Direction of Association	*p*-Value
Lives with father	Higher PD scores when NOT living with father	0.015
Maternal intolerance to light/noise	Higher PD scores when present	<0.001
Maternal sadness	Higher PD scores when present	<0.001
Maternal fatigue	Higher PD scores when present	0.027
Maternal body pain	Higher PD scores when present	0.001
Maternal insomnia	Higher PD scores when present	0.001
Difficulty falling asleep	Higher PD scores when present	0.001
Early morning awakenings	Higher PD scores when present	0.040
Income class (ABEP)	No significant difference	>0.05
Parental education	No significant difference	>0.05

**Table 8:** QFVI 3 Descriptive Scores.

Domain	Mean Score
General Functioning	59.4
Emotional Impact	62.9
Social Interaction	66.2
Daily Activities	70.4
Family Burden	69.0
Parental Concerns	75.6

**Table 9:** QFVI 7 Descriptive Scores.

Domain	Mean Score
General Functioning	64.2
Emotional Impact	68.6
Social Interaction	71.0
Daily Activities	73.9
Family Burden	76.4
Parental Concerns	78.3

**Table 12:** Exploratory Predictors of Parental Stress.

Predictor Variable	Univariate Association (*p*-Value)	Multivariable β	Multivariable *p*-Value
Maternal depressive symptoms	<0.001	+0.41	<0.01
Socioeconomic class	<0.01	−0.25	0.03
Lives with father of child	<0.05	+0.22	0.04
QFVI total score	<0.05	−0.20	0.04
Child age	>0.05	n.s.	-
Maternal age	>0.05	n.s.	-
Physical activity	<0.05 (univariate)	n.s.	-

n.s. = non-significant.

## Data Availability

The datasets generated and analyzed during the current study have been deposited in the Harvard Dataverse repository and will be made publicly available upon acceptance of this manuscript. After publication, all dataset files will be accessible under a CC0 1.0 Universal Public Domain Dedication. Access to identifiable or sensitive raw data remains restricted in accordance with ethical regulations and with the approval granted by the Research Ethics Committee of the Federal University of Goiás (CAAE 90833718.2.0000.5078).

## Abbreviations

The following abbreviations are used in this manuscript:

PSI-4	Parenting Stress Index, Fourth Edition
CD	Child Domain (PSI-4)
PD	Parent Domain (PSI-4)
LS	Life Stress (PSI-4)
QFVI-3	Quality of Family Vision Impact, version for ages 0–3 years
QFVI-7	Quality of Family Vision Impact, version for ages 3–7 years
CVFQ	Children’s Visual Function Questionnaire
ABEP	Brazilian Association of Research Companies (Socioeconomic Classification)
DP	Depression Subscale (PSI-4 Parent Domain)
HE	Health Subscale (PSI-4 Parent Domain)
AD	Adaptability Subscale (PSI-4 Child Domain)
DE	Demandingness Subscale (PSI-4 Child Domain)
UFG	Federal University of Goiás
IRB	Institutional Review Board

## Appendix A. STROBE Checklist

This appendix contains the complete STROBE (Strengthening the Reporting of Observational Studies in Epidemiology) checklist for cross-sectional studies. The checklist summarizes how each reporting item was addressed in the present study, ensuring transparency, methodological rigor, and adherence to international standards for observational research reporting.

**Table A1:** STROBE Checklist.

Item	STROBE Recommendation	Description	Location in Manuscript
1	Title and abstract	Indicate study design in title or abstract; provide balanced summary	Title page; Abstract
2	Background/rationale	Explain scientific background and rationale	Introduction
3	Objectives	State specific objectives, hypotheses	Introduction
4	Study design	Present key elements early	Methods—Study Design
5	Setting	Describe setting, locations, relevant dates	Methods—Setting
6	Participants	Give eligibility criteria and selection methods	Methods—Participants
7	Variables	Clearly define all outcomes, exposures, confounders	Methods—Variables
8	Data sources/measurement	Provide measurement details for each variable	Methods—Instruments (PSI-4, QFVI)
9	Bias	Describe efforts to address potential sources of bias	Methods—Data Collection; Limitations
10	Study size	Explain how sample size was determined	Methods—Participants
11	Quantitative variables	Explain handling of quantitative variables and groupings	Methods—Statistical Analysis
12	Statistical methods	Describe all methods including control for confounding, subgroup analyses	Methods—Statistical Analysis
13	Participants flow	Report numbers at each stage	Results—Flow of Participants
14	Descriptive data	Provide participant characteristics	Results—Section 3.1
15	Outcome data	Report numbers of outcome events or summary measures	Results—Section 3.2, Section 3.3, Section 3.4, Section 3.5 and Section 3.6
16	Main results	Provide unadjusted and adjusted estimates, confidence intervals	Results—Tables
17	Other analyses	Report subgroup and sensitivity analyses	Results—Group Comparisons; Correlations
18	Key results	Summarize key findings with respect to study objectives	Discussion—Opening paragraph
19	Limitations	Discuss limitations of the study	Discussion—Limitations paragraph
20	Interpretation	Give cautious overall interpretation	Discussion
21	Generalisability	Discuss external validity	Discussion—Generalization Notes
22	Funding	Describe funding sources and roles	Funding Statement
